# Multi-view radiomics and deep learning modeling for prostate cancer detection based on multi-parametric MRI

**DOI:** 10.3389/fonc.2023.1198899

**Published:** 2023-06-28

**Authors:** Chunyu Li, Ming Deng, Xiaoli Zhong, Jinxia Ren, Xiaohui Chen, Jun Chen, Feng Xiao, Haibo Xu

**Affiliations:** ^1^ Department of Radiology, Zhongnan Hospital of Wuhan University, Wuhan, China; ^2^ GE Healthcare, Shanghai, China

**Keywords:** prostate cancer, multi-parametric MRI, multi-view radiomics, deep learning, nomogram

## Abstract

**Introduction:**

This study aims to develop an imaging model based on multi-parametric MR images for distinguishing between prostate cancer (PCa) and prostate hyperplasia.

**Methods:**

A total of 236 subjects were enrolled and divided into training and test sets for model construction. Firstly, a multi-view radiomics modeling strategy was designed in which different combinations of radiomics feature categories (original, LoG, and wavelet) were compared to obtain the optimal input feature sets. Minimum-redundancy maximum-relevance (mRMR) selection and least absolute shrinkage selection operator (LASSO) were used for feature reduction, and the next logistic regression method was used for model construction. Then, a Swin Transformer architecture was designed and trained using transfer learning techniques to construct the deep learning models (DL). Finally, the constructed multi-view radiomics and DL models were combined and compared for model selection and nomogram construction. The prediction accuracy, consistency, and clinical benefit were comprehensively evaluated in the model comparison.

**Results:**

The optimal input feature set was found when LoG and wavelet features were combined, while 22 and 17 radiomic features in this set were selected to construct the ADC and T2 multi-view radiomic models, respectively. ADC and T2 DL models were built by transferring learning from a large number of natural images to a relatively small sample of prostate images. All individual and combined models showed good predictive accuracy, consistency, and clinical benefit. Compared with using only an ADC-based model, adding a T2-based model to the combined model would reduce the model’s predictive performance. The ADCCombinedScore model showed the best predictive performance among all and was transformed into a nomogram for better use in clinics.

**Discussion:**

The constructed models in our study can be used as a predictor in differentiating PCa and BPH, thus helping clinicians make better clinical treatment decisions and reducing unnecessary prostate biopsies.

## Introduction

As the most common malignancy of the male genitourinary system, prostate cancer (PCa) has become the second leading cause of cancer death in men (1, 2). Early diagnosis of PCa allows patients to choose the best treatment options, which can improve treatment effectiveness, reduce mortality, and improve quality of life. However, it is not easy to detect PCa because there are many similar clinical symptoms between PCa and other prostate diseases, such as benign prostatic hyperplasia (BPH), prostatitis, urinary tract infection, cystitis, and urethral stricture (3). Traditional methods (4) for detecting PCa mainly include the serum prostate-specific antigen (PSA) test, digital rectal examination (DRE), and a routine method of puncture biopsy guided by transrectal ultrasound (TRUS). However, these methods have been reported to be of low sensitivity and specificity (5) and/or may cause infection, bleeding, and pain (6, 7). Many patients are undergoing unnecessary biopsies for BPH. Furthermore, possible false-negatives remain a problem for TRUS-guided biopsies (8). Therefore, a noninvasive and exact diagnosis method for PCa is of great significance.

MRI is considered one of the most promising imaging methods for PCa detection because of its noninvasiveness and the rich information about soft tissue contrast contained in different sequences (9, 10). The latest Prostate Imaging Reporting And Data System (PI-RADS) v2.1 recommends bi-parameters, T2-weighted imaging (T2WI) and diffusion-weighted imaging (DWI), for PCa detection. DWI is one of the most important image sequences in MRI and quantifies the diffusion motion characteristics of water molecules in tissues through the apparent diffusion coefficient (ADC), which can provide useful diagnostic information such as cell density, cell membrane integrity, and intercellular substances in tissues and helps to distinguish cancerous and non-cancerous lesions (11). However, at present, MRI interpretation relies on radiologists with specialized training and subjective clinical experience, which lack quantitative evaluation and objective tools.

With the development of computer science, image processing technology and artificial intelligence (AI) method has been more and more widely used in the precise diagnosis and treatment of diseases, which extends research ideas and provides effective tools for the early diagnosis, treatment, and prognosis analysis of diseases. The connotative characteristics of disease can be discovered through in-depth data mining (12, 13). Radiomics is a quantitative image analysis method that can extract high-throughput features from medical images to quantify characteristics of major diseases such as tumors, and shows great advantages in tumor phenotype typing, treatment decision, and prognosis analysis (14–17). Compared to the radiomic method, deep learning methods (18) can adaptively learn and extract useful feature information from a large amount of data. The constructed multi-layer deep neural network model can achieve high classification and prediction accuracy for clinical use. So far, both methods have been applied to prostate disease-related domains, including PCa detection, grading, tumor habitats (19, 20), and so on. However, combining these two methods to handle prostate clinical problems based on the use of multi-parametric MR images has not been reported in the previous literature.

Thus, in the present study, a multi-parametric MR image prediction model based on radiomics and a deep learning method was developed, aiming to discriminate PCa from BPH. With this non-invasive early diagnosis, patients with PCa can receive timely treatment and management, while patients with BPH can avoid unnecessary biopsies.

## Method

This study was a retrospective study that was implemented at Zhongnan Hospital of Wuhan University, Wuhan, China.

### Patients

All patients were searched in the Picture Archiving and Communications System (PACS) of Zhongnan Hospital of Wuhan University between January 2018 and December 2021. These enrolled patients underwent multi-parameter MRI image acquisition before the prostate pathology examination and were pathology-proven to have PCa or BPH. The exclusion criteria were a) patients received endocrine therapy, radiotherapy, cryotherapy, or surgery prior to MRI scanning; b) MRI images are of poor quality because of motion artifacts, metal artifacts, or susceptibility artifacts; and c) the clinical records, such as age and/or PSA, were incomplete. It should be noted that the patients having both diseases were categorized as PCa groups.

### Pathological examination

All patients underwent a TRUS-guided 13-core prostate biopsy. The pathological results were evaluated using Gleason grading and scoring. The Gleason score is the sum of the two most widely used levels of cell structure, such as 3 + 4, 4 + 3, etc., and is commonly used for PCa diagnosis and treatment strategy decision-making (21).

### Data flowchart

**Figure 1:**
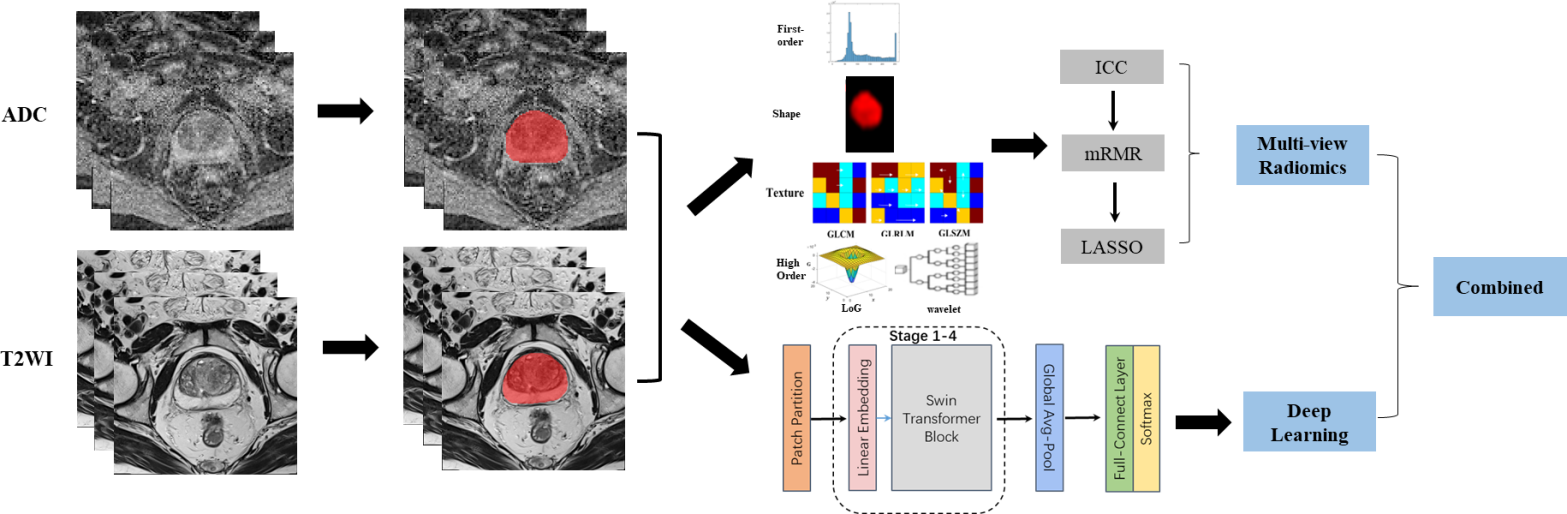
Image analysis flow chart for this study. From left to right, the complete image analysis process could be divided into three parts: image acquisition, region of interest (ROI) segmentation, and predictive modeling. Two strategies were adopted in the predictive modeling. Multi-view radiomics strategy(top-right) contained radiomics features extraction, feature reduction and statistical modeling while deep learning strategy (right-bottom) contained a 4-stages Swin Transformer architecture design. Finally, The models constructed using different strategy and based on different modes of images were combined and compared for clinical use.

The workflow of this study mainly contained two parts: multi-view radiomics and deep learning. As shown in **Figure 1**, the procedures before these two methods were image acquisition and segmentation. The multi-view radiomics modeling consists of four steps: 1) radiomics feature extraction, 2) optimal input feature set combination, 3) feature selection, and 4) statistical modeling. A multi-stage Swin transformer architecture is designed for deep learning modeling. In this study, both modeling strategies were adopted for the ADC maps and T2WI images, respectively. Finally, all constructed models were randomly combined and compared with each other to find the best way to distinguish PCa patients from BPH patients.

All 236 subjects were split into a training set (164, 70%) and a test set (72, 30%) using a stratified random sampling method, in which the samples are stratified according to the ratio between different groups of PCa and BPH and then randomly sampled. The samples from the training set were used for model construction, while the performance of the models was verified and compared using the training set and the test set, respectively.

### Image acquisition and processing

MRI examinations of all patients were performed using the same 1.5T MRI scanner (Aera, Siemens Healthcare, Erlangen, Germany), with an 18-channel body phased array coil above the pelvis and a spine coil under the pelvis. Patients defecated and urinated before the MRI examination to ensure high image quality. MRI acquisition sequences include transverse, coronal, and sagittal T2-weighted imaging (T2WI), transverse fat-suppressed T2-weighted imaging, and transverse diffusion-weighted imaging (DWI). DWI includes two b-values of 0 and 1,500. An apparent diffusion coefficient (ADC) map was generated after DWI completion in the scanner. In this study, only transverse T2WI images and ADC maps are included for the data analysis.

The protocol parameters of T2WI images are as follows: repetition time (TR) = 6,910 ms, echo time (TE) = 112 ms, thickness = 3 mm, field of view (FOV) = 180 × 180 mm, number of excitations (NEX) = 3, matrix = 320 × 320, pixel spacing = 0.6 × 0.6 mm, flip angle = 160. The parameters of DWI: repetition time (TR) = 4,620 ms, echo time (TE) = 58 ms, thickness = 3.5 mm, field of view (FOV) = 200 × 200 mm, number of excitations (NEX) = 2, matrix = 116 × 116, pixel spacing = 1.7 × 1.7 mm, flip angle = 180.

First, the linear interpolation method was used to resample images of the same sequence to the same voxel size (0.6 mm ∗ 0.6 mm ∗ 3 mm for T2WI images, 1.7 mm ∗ 1.7 mm ∗ 3.5 mm for ADC maps). Then the whole prostate in the images was segmented as the volumes of interest (VOI) using ITK-SNAP software (http://www.itksnap.org/). Manual segmentation of the VOIs was performed slice by slice on transverse T2WI images and ADC maps, respectively. The procedure was completed independently by two radiologists with more than 10 years of experience in the genitourinary system (MD and JR) to ensure the repeatability and reliability of the results, which can be evaluated using inter-class and intra-class correlation coefficients (ICC) (details in **Supplementary Methods**). The Pyradiomics package (https://pyradiomics.readthedocs.io/en/latest/) was used to calculate the radiomic features of the VOIs and resulted in 1,561 radiomic features (details in **Supplementary Methods**) for the prostate of each patient.

### Feature reduction and radiomics model construction

Feature reduction in this study contained three steps: 1) ICC analysis was used to screen the radiomic features with better reliability and repeatability; 2) the maximum relevance minimum redundancy (mRMR) algorithm was used to select an optimal subset from the inputted features that maximized their relevance to the classification variable while minimizing redundancy between features, to reduce computational cost and improve predictive performance. The parameter of optimal feature subset size in mRMR (*N_mRMR_*) was determined using grid search and bootstrapping with 100 replicates; 3) the least absolute shrinkage and selection operator (LASSO) (22) model was used for further feature selection. LASSO was a linear regression method whose basic idea was to penalize unimportant variables in the model by adding an L1 regularization term, thus making the model simpler and sparser and reducing the risk of overfitting. In this study, the LASSO method used 10-fold cross-validation and minimum prediction error criteria. In the results, the features with non-zero coefficients were retained in the radiomics model construction with a multi-variate logistic regression method. In this study, two individual radiomics models, ADCScore and T2Score, were constructed based on the ADC maps and T2WI images, respectively.

The high dimension complexity and possible inner collinearity caused by many features could easily lead to overfitting of the established model. To eliminate or alleviate the effects of this issue, a multi-view radiomics (**Figure S1**) modeling scheme was designed (23). Firstly, three categories of radiomics features (C1: original, C2: LoG, and C3: wavelet; detailed in **Supplementary Methods**) were used in model construction independently or in random combination, which resulted in seven models with different input radiomics feature categories (C1, C2, C3, C1 + C2, C1 + C3, C2 + C3, and C1 + C2 + C3). Then, these models were compared to determine the most appropriate input radiomics feature category or category combination in radiomics model construction.

### Deep learning model construction

**Figure 2:**
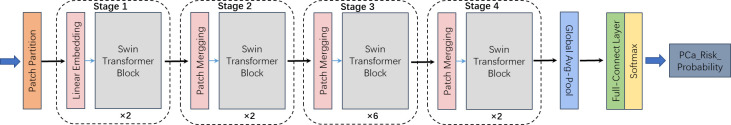
The architecture of the 4-stages Swin Transformer used in deep learning modeling.

In this study, a DL network architecture called Swin Transformer (24, 25) was designed to construct a DL model for the prediction of PCa. **Figure 2** illustrates the architecture of the Swin Transformer, which is also detailed in **Supplementary Methods**. Firstly, a three-dimensional rectangular bounding box was defined in the image according to the prostate VOI to ensure that the entire prostate was completely contained in the bounding box. Then, each image slice within the bounding box was then resampled to 224 × 224 pixels using bilinear interpolation. In this study, three adjacent image slices in a bounding box were combined into a three-channel image, which was used as the input of the DL model, and the model output was the PCa risk probability. To obtain a more stable prediction, all the three-channel images of the prostate in a bounding box were inputted into the DL model, and the mean value of all output PCa risk probabilities was calculated as the final output score of this DL model.

The transfer learning technique (26) was used in the development of the DL model. The pre-training model was trained based on many natural images in the public dataset of ImageNet. Then the final model was fine-tuned using our prostate MRI images. In this study, two individual DL models, ADCDLScore and T2DLScore, were built based on the ADC maps and T2WI images, respectively.

### Combined models and nomogram construction

As independent risk factors, the four constructed image models (ADCScore, T2Score, ADCDLScore, and T2DLScore) were combined using a multivariate logistic regression approach, resulting in five different combined models (RadScore, DLScore, ADCCombinedScore, T2CombinedScore, and CombinedScore; see **Table 1** for details). After model performance comparison, the combined model with the best predictive performance was transformed into a nomogram for better visualization, interpretation, and clinical use.

**Table 1:** Construction details for different combined models.

Models	Variables	methods
RadScore	ADCScore**+** T2Score	Logistic Regression
DLScore	ADCDLScore+ T2DLScore	Logistic Regression
ADCCombinedScore	ADCScore+ ADCDLScore	Logistic Regression
T2CombinedScore	T2Score+T2DLScore	Logistic Regression
CombinedScore	ADCScore+ ADCDLScore+ T2Score+ T2DLScore	Logistic Regression

### Model comparison and validation

A series of metrics were used to evaluate and compare the diagnostic performance of the models. First, the predictive accuracy of all constructed models was evaluated using four receiver operator characteristic (ROC)-related indicators: area under the curve (AUC), accuracy, sensitivity, and specificity, and the Delong test was used to compare whether the difference in prediction accuracy between the models was significant. Then the calibration curve and Hosmer–Lemeshow test evaluated the consistency between the model predictions and actual observations. Finally, decision authority was used to evaluate the net benefit of using the model for diagnosis in clinical practice.

### Statistics

In this study, we implemented the Swin Transformer DL model in the Pytorch framework using Python 3.7.0. The R 4.0.5 environment was used to construct the multi-view radiomics model and the combined models, specifically: the “mRMRe” and “glmnet” packages were used to realize the screening and further selection of radiomics features, respectively, and the “pROC” package was used to draw the ROC curve and compute relevant indicators for model validation and comparison.

## Results

### Patient characteristics

As shown in **Figure S2**, a total of 236 subjects (PCa: 100 and BPH: 136) were included in this study. The stratified random sampling method was then used to divide all subjects into a training set (164) and a test set (72). **Table 2** shows the detailed demographic characteristics of all subjects and their distribution in the training and test sets.

**Table 2:** Patients demographics for training and test set.

Characteristic	Training set (164)		*p*-value	Test set (72)		*p*-value
	Ca(n=69)	BPH (n=95)		Ca (n=31)	BPH (n=41)	
Age	72.81±7.14	69.86±7.53	0.012	71.06±7.95	69.80±8.21	0.514
PV (cm^3^)	46.37±42.41	65.79±44.03	0.005	43.76±36.80	58.56±27.41	<0.001
TPSA(ng/mL)	24.08±21.67	13.32±10.95	<0.001	25.74±24.27	13.11±7.13	0.008
FPSA(ng/mL)	3.28±3.34	2.76±3.92	0.363	3.09±2.87	2.41±1.73	0.247
Gleason Score						
3+3=6	21(30.43%)	–	–	9(29.03%)	–	–
3+4=7	6(8.70%)			4(12.90%)		
4+3=7	11(15.94%)			3(9.68%)		
3+5=8	5(7.25%)			2(6.45%)		
5+3=8	3(4.35%)			1(3.23%)		
4+4=8	11(15.94%)			4(12.90%)		
4+5=9	2(2.90%)			4(12.90%)		
5+4=9	5(7.25%)			3(9.68%)		
5+5=10	5(7.25%)			1(3.23%)		

PV, Prostate Volume; TPSA, total prostate specific antigen; FPSA, free prostate specific antigen.

### Image analysis and model construction

**Figure 3:**
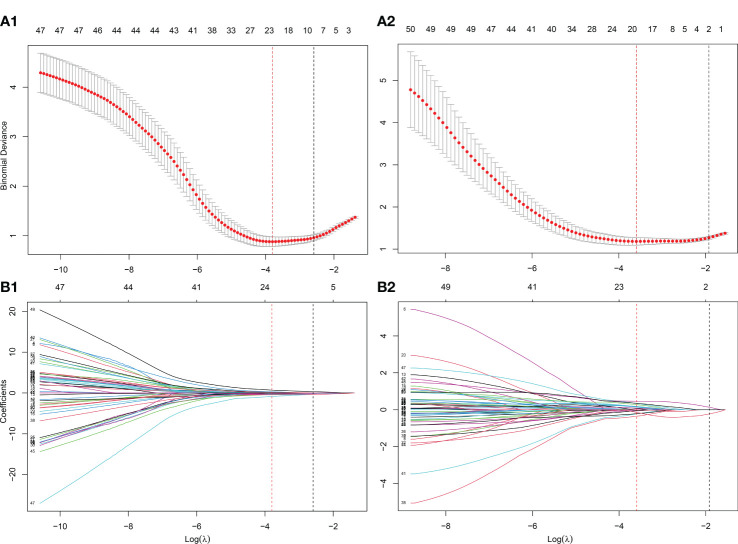
Further screening of radiomics features using the LASSO method. **(A)** The determination of the key parameter (penalty coefficient: λ) in the LASSO model using 10-fold cross-validation. Two rules resulted in two λ values (λ_min_: when the predicion error reached the minimum and λ_1se_: the value within one standard error from the minimum) and two vertical dashed lines at their position were drawn. λ_min_ was adopted in the feature selection of LASSO in this study; **(B)** Feature coefficients profiles as the λ value changes. According to the 10-fold cross-validation in **(A)**, the features with non-zero coefficients were further selected at the position of λmin. (A1-B1) λ_min_=0.0224 with log( λ_min_)=-3.7991 was selected for the ADC radiomics model (ADCScore) construction, in which 22 features with nonzero coefficients were finally selected. (A2-B2) λ_min_=0.0275 with log( λ_min_)=-3.5953 was selected for the T2 radiomics model (T2Score) construction, in which 17 features with nonzero coefficients were finally selected.

As shown in **Figure 1**, the whole prostate in the ADC maps and T2WI images was manually segmented as VOIs by two radiologists. Then, a total of 1,561 quantitative features (C1: 106; C2: 701; C3: 754) were extracted from the VOIs. A total of 1,210 features (C1: 97; C2: 369; C3: 744) in the ADC map and 1,206 features (C1: 104; C2: 370; C3: 732) in the T2 image showed good repeatability and stability (ICC >0.8) and were retained for subsequent radiomics modeling. Through the predictive performance comparison of the models constructed based on different input feature categories (**Table 3**), C1 + C3 were selected as the optimal input feature categories for the radiomics modeling in this study. The number of features was first reduced to 50 using the mRMR method and then input into the LASSO model, which finally resulted in 22 and 17 features (**Figure 3**) for ADC and T2 radiomics model construction, respectively. The final selected features were listed in **Tables S1**, **S2** in detail. ADCScore and T2Score were the output probability scores for the corresponding radiomics model constructed based on ADC map and T2 images, respectively, which can be used for model performance evaluation and further combined model construction.

**Table 3:** AUC comparison of different radiomics model inputted with different subcategorizing radiomics features for the ADC maps and T2WI images.

	ADC				T2			
	Rad_train	Rad_test	*N_mRMR*	*N_LASSO*	Rad_train	Rad_test	*N_mRMR*	*N_LASSO*
C1	0.969	0.926	50	24	0.8805	0.753	100	16
C2	0.8863	0.8442	50	13	0.8321	0.7278	50	8
C3	0.9464	0.9315	100	17	0.8853	0.7333	50	17
C1+C2	0.9448	0.9339	200	21	0.8704	0.7467	50	14
C1+C3	**0.9684**	**0.9355**	**50**	**22**	**0.895**	**0.764**	**50**	**17**
C2+C3	0.9509	0.919	100	17	0.9099	0.7404	50	25
C1+C2+C3	0.9653	0.9182	50	21	0.8856	0.7545	200	11

C1: original radiomics features; C2: radiomics features for the images after LoG transformation; C3: radiomics features for the images after wavelet transformation; N_mRMR: the number of features retained using mRMR method; N_LASSO: the number of features retained using LASSO method.

Transfer learning was employed in DL model construction: firstly, the Swin Transformer model was pre-trained based on millions of natural images in the ImageNet dataset; subsequently, secondary training (fine-tuning) was performed based on the ADC maps and T2 images of patients in our training set to make the DL model well-suited to in the prediction task of this study. ADCDLScore and T2DLScore were expressed as the output probability scores of the DL models and could be used in model performance evaluation and further combined model construction.

As shown in **Tables S3–S7**, five combined models (RadScore, DLScore, ADCCombinedScore, T2CombinedScore, and CombinedScore) were finally constructed using the logistic regression model.

### Model validation and comparison

**Figure 4:**
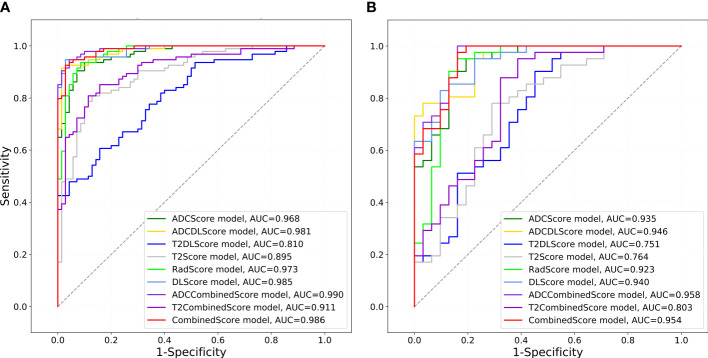
ROCs drawn for different models. The gray diagonal lines in **(A)** training set and **(B)** test set indicate an AUC value of 0.5, which means the prediction result of completely random.

**Figure 5:**
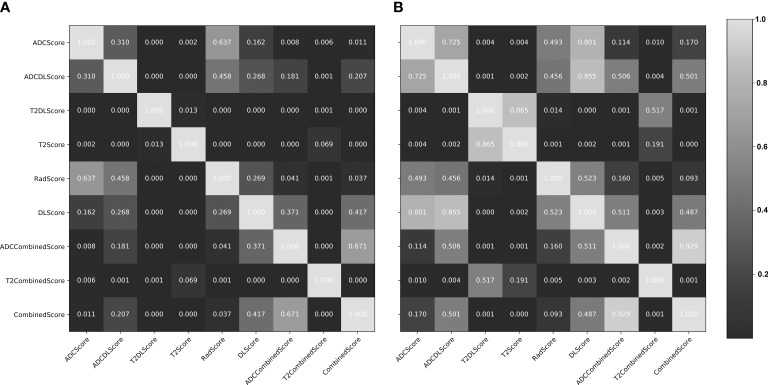
Delong test results (*p*-value maps) between different constructed models. **(A)** training set; **(B)** test set. The closer the map color is to black, the smaller the p-value and the more significant the performance difference between the models; the closer the map color is to white, the larger the p-value and the more insignificant performance difference between the models.

**Figure 4** shows the ROC of the nine models constructed in this paper, and **Table 4** lists their ROC-related indicators (accuracy, sensitivity, specificity, and their 95% confidence interval) in detail, which fully assesses the prediction accuracy of the constructed models. The *p*-value map shown in **Figure 5** further illustrated the predictive accuracy difference between different models, and *p <*0.05 indicated that the difference between the corresponding two models was significant. The calibration curves with Hosmer–Lemeshow test results shown in **Figures S3**, **S4** exhibited the consistency between the model prediction and actual observation for all models.

**Table 4:** Detailed ROC-related metrics for all constructed models.

Models		AUC	Accuracy(95%CI)	Sensitivity(95%CI)	Specificity(95%CI)
ADCScore	train	0.968	0.921(0.918-0.924)	0.9(0.892-0.908)	0.936(0.931-0.941)
	test	0.935	0.861(0.852-0.871)	0.806(0.781-0.831)	0.902(0.888-0.917)
ADCDLScore	train	0.981	0.945(0.942-0.948)	0.986(0.982-0.989)	0.915(0.909-0.921)
	test	0.946	0.819(0.809-0.830)	0.839(0.815-0.862)	0.805(0.786-0.824)
T2DLScore	train	0.810	0.707(0.702-0.713)	0.843(0.833-0.853)	0.606(0.596-0.617)
	test	0.751	0.639(0.626-0.652)	0.774(0.748-0.801)	0.537(0.513-0.56)
T2Score	train	0.895	0.835(0.831-0.840)	0.871(0.862-0.881)	0.809(0.8-0.817)
	test	0.764	0.750(0.738-0.762)	0.710(0.681-0.738)	0.780(0.761-0.8)
RadScore	train	0.973	0.927(0.924-0.930)	0.914(0.906-0.922)	0.936(0.931-0.941)
	test	0.923	0.875(0.866-0.884)	0.839(0.815-0.862)	0.902(0.888-0.917)
DLScore	train	0.985	0.957(0.955-0.960)	0.971(0.967-0.976)	0.947(0.942-0.951)
	test	0.940	0.875(0.866-0.884)	0.774(0.748-0.801)	0.951(0.941-0.962)
ADCCombinedScore	train	0.990	0.951(0.949-0.954)	0.957(0.951-0.963)	0.947(0.942-0.951)
	test	0.958	0.889(0.880-0.897)	0.839(0.815-0.862)	0.927(0.914-0.939)
T2CombinedScore	train	0.911	0.841(0.837-0.846)	0.886(0.877-0.895)	0.809(0.8-0.817)
	test	0.803	0.694(0.682-0.707)	0.677(0.648-0.707)	0.707(0.686-0.729)
CombinedScore	train	0.986	0.945(0.942-0.948)	0.971(0.967-0.976)	0.926(0.92-0.931)
	test	0.954	0.875(0.866-0.884)	0.839(0.815-0.862)	0.902(0.888-0.917)

Youden criterion: The best operating point of the ROC was chosen at the point whose Youden index (Sensitivity+Specificity-1) is maximal.

Overall, except for T2Score (AUC = 0.751), T2DLScore (AUC = 0.764), and T2CombinedScore (AUC = 0.803), all other six models showed excellent prediction accuracy (AUC >0.92 for the test set in **Figure 4** and **Table 4**). The calibration curves and Hosmer–Lemeshow test (p >0.05 in **Figures S2**, **S3**) showed good uniformity between their observed and predicted values for all models. Combining the results of the Delong test (**Figure 5**), we found during the modeling process:

(1) When only ADC maps were used, the DL strategy (ADCDLScore) was better than the radiomics strategy (ADCScore), but the difference between them was not significant; when only T2WI images were used, the radiomics strategy (T2Score) significantly outperformed the DL strategy (T2DLScore).

(2) The three T2WI image-based models, T2Score, T2DLScore, and T2CombinedScore, were significantly worse than the other models in prediction performance;

(3) Compared with using only the ADC-based model, adding the T2-based model to the combined model would reduce the model’s predictive performance, but this performance degradation was not significant;

(4) The ADCCombinedScore model showed the best predictive performance among all, but the difference between it and other ADC map-based models was not significant.

### Clinical use and explanation

**Figure 6:**
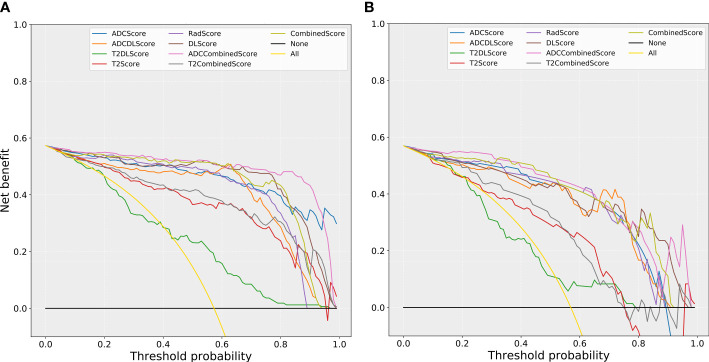
The comparison of decision curves for the different models. **(A)** training set; **(B)** test set. Treat-all strategy: All patients were diagnosed as positive; treat-none: All patients were diagnosed as negative.

**Figure 6** compares the net benefit obtained when the constructed models were used in the clinic:

(1) All models (except the T2DL model) could bring more benefits than the “treat all” or “treat none” strategies used for almost all the risk thresholds (except the “treat none” strategies at some of the high threshold intervals).

(2) All models based only on T2WI images (T2Score, T2DLScore, and the T2CombinedScore models) obtained less benefit than the other models for almost all the risk thresholds (except some of the high threshold intervals).

**Figure 7:**
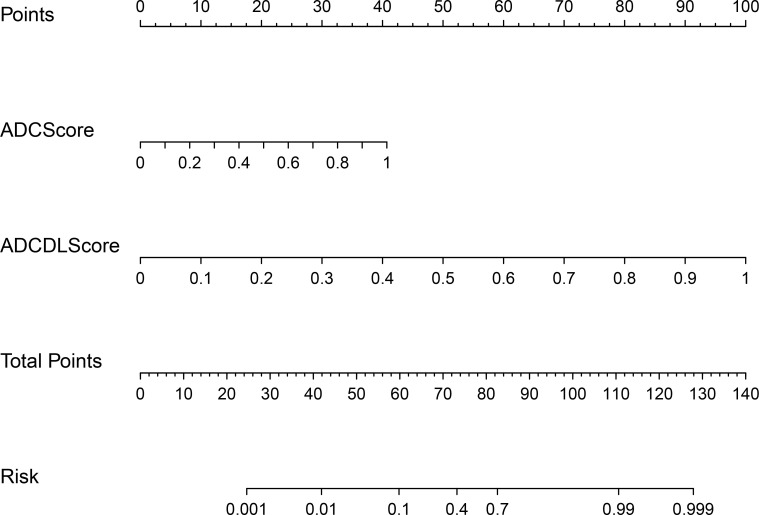
CombinedScore nomogram. The CombinedScore nomogram constructed by combining the ADCScore, ADCDLScore, T2Score and T2DLScore models, which was used as independent risk factors.

**Figure 7** shows the nomogram drawn based on the ADCCombinedScore model (best predictive performance), in which ADCScore and ADCDLScore were represented as two independent risk factors in the prediction of PCa. By adding the individual scores corresponding to ADCScore and ADCDLScore, the total score could finally be used to quantitatively predict the risk probability of PCa.

## Discussion

In this study, we develop and validate a multi-parameter prostate MRI-based model for noninvasive, quantitative prediction of PCa. The ADCCombinedScore model showed better predictive performance in distinguishing PCa with BPH than any other model (ADCScore, T2Score, ADCDLScore, T2DLScore, RadScore, DLScore, T2CombinedScore, and CombinedScore). Among all, the ADCCombinedScore model showed the highest predictive accuracy (AUC = 0.958 in the test set) and was finally transformed into a nomogram for better clinical use, in which ADC radiomics and deep learning scores were used as independent risk factors.

Until now, many radiomics and deep learning methods have been used for prostate detection and diagnosis. Most radiomics studies developed radiomics models by combining different feature reduction methods and machine learning models. Wu et al. (27) built an LR model to evaluate the quantitative image features for the diagnosis of transition zone PCa and achieved high predictive performance (AUC = 0.989). However, there is no independent validation set to guarantee the reliability of their results. The radiomics model constructed by Chen et al. (28) obtained the highest predictive performance (AUC = 0.985, 0.982, and 0.999) on T2, ADC, and their combination T2&ADC. However, their ROIs were manually depicted along the boundaries of the lesion, slice by slice, in reference to the pathological findings of the biopsy. This histological–radiological matching was tedious and difficult to perform, which inevitably introduced bias and limited the stability, repeatability, and clinical utility of the constructed model. Ji et al. (29), He et al. (30), and Xu et al. (31) also constructed radiomics models with high predictive performance, respectively. The AUC of their models ranged from 0.86 to 0.93 but was lower than the AUC of our multi-view model (AUC = 0.958). This may be due to the adoption of multi-view schemes for radiomic features to avoid overfitting to some extent and the complementarity of radiomic methods and deep learning methods in mining image information at different depths. Hu et al.’s (32) work showed that the type and size of samples have a great influence on the performance of the DL models established using transfer learning techniques. The transfer models learned from disease-related images perform better than those learned from natural images. This provides us with ideas to improve the performance of deep learning modeling in the future.

In this study, we adopted two imaging modeling strategies: multi-view radiomics and deep learning. Overfitting may occur when using many radiomic features. Even if we utilized mRMR and LASSO for feature selection, collinearity between features and their high dimensionality may have impaired model performance (33). To address this issue, we designed a multi-view radiomics strategy that tried to determine optimal input feature subsets by subcategorizing and combining different features and could finally improve the performance of a radiomics model. The comparison results also confirmed that the final constructed multi-view model (C1 + C3) was better than the single-view model (C1 + C2 + C3). In general, higher-order features (LoG and wavelet) could provide more diagnostic information and thus play a more important role in radiomics modeling (34). However, results in this study showed that not every combination of high-order features could always result in a high-performance model. For example, adding LoG features to the combined model reduced the model’s performance. This may be because the higher the level of features, the easier it is for various complex linear or nonlinear correlations to appear between them (35). Although several feature reduction strategies and methods (multi-view radiomics strategy, mRMR, and LASSO) were used in this study, it is still a difficult problem to solve properly, and more research and exploration in this area are needed in the future. Multi-layer convolution and filtering of images in deep learning methods could generate ultra-high-dimensional disease-related features. These features were generally difficult to interpreted clinically but showed a high correlation with patient grouping labels. The constructed models based on these features usually show a high clinical application value. In this study, multi-view radiomics and deep learning methods were used at the same time to extract a variety of interpretable or unexplainable multi-dimensional imaging features for the prostate, which could comprehensively reflect the heterogeneity of prostate lesions in images and was also the basis for establishing a PCa risk prediction or diagnosis model. In fact, radiomics and deep learning methods could provide different perspectives on the data level for the modeling of this paper. The dimensions and depths of the image feature information excavated by them were not the same: the radiomics method provided a moderate depth level of the image feature information, while the deep learning method provided a much deeper level. Therefore, modeling using a combination of radiomics and deep learning methods was also a multi-perspective strategy.

It is worth noting that the segmentation method used in this study was manual segmentation of the whole prostate. Most current studies (27–31) only focus on the segmentation of the lesion, which leads to the predictive modeling of lesion classes and limits the clinical usability of developing models. In real-life application scenarios, many occult lesions in the prostate and their boundaries are difficult to distinguish with the naked eye, which could be solved by including the whole prostate in the modeling. In addition, whole-prostate segmentation is more suitable for patients with both PCa and BPH. As described in the *Methods* section of this study, we categorized such patients as PCa patients during the modeling analysis.

Most published studies suffer from the problem of data selection bias. Many studies only investigated patients with clinically significant PCa of Gleason Score 7 or greater, ignoring clinically insignificant PCa of Gleason Score 6. In this study, 30 clinically insignificant cases make up 30% of PCa patients, enriching the sample variety. The finding may be more reliable for a wider population of patients with PCa. Prostate tumors have poorly defined margins, which makes manual segmentation challenging. Furthermore, clinically insignificant PCa generally have low-volume cancerous tissue and may have no obvious lesions, leading to the absence of tumors for radiologists. As an improvement to this problem, we segmented the whole prostate gland instead of lesions in this study, ensuring stability and reproducibility. Most studies have used 3T MRI scanners for data acquisition, and studies using 1.5T MRI systems for data acquisition are currently very limited. This study could provide a supplement to the present research data for 1.5T MRI, and the results show that 1.5T magnetic resonance scanners also have high application value in the detection and identification of PCa.

T2WI radiomics and DL models were all excluded from the final model construction and achieved the best predictive performance. Compared to ADC maps, T2WI has relatively little effect on the predictive model, which is consistent with previous work (36). Cancerous tissue shows low signal intensity on T2WI, which is the same for BPH, leading to a lack of specificity for T2WI. DWI and ADC maps can reveal water molecules diffusion in tissues, which indicates a possible change in cell density and/or intercellular substance in prostate tissues, while T2WI only provides structural information. Previous studies have proven the usefulness of ADC maps for evaluating PCa (37, 38).

To comprehensively evaluate the research quality of our study, we did a self-assessment with the Radiomics Quality Score (RQS) (39), and obtained an RQS of 15 (41.67%) (details in **Table S8**), which was higher than the average level of the radiomics studies on prostate MRI (23% ± 13%) (40) and the radiomics studies in general (median = 21%, IQR = 11.50) (41). As seen from the items without any scores in **Table S5**, we found the main problem of this study was that the number of subjects included in this study was limited, providing limited statistical power. More external data from multiple centers are needed to validate our results and conclusions. Furthermore, validation using prospective data can provide the highest level of evidence supporting the clinical validity and usefulness of the constructed models.

Besides, there are the following limitations in this study: Firstly, PCa staging is not involved in this study. Although the Gleason score of every histologic core in a biopsy is acquired, it is difficult to locate the exact region on MRI images. PCa staging is important and requires investigation. MRI-targeted biopsy is the trend for the future. Secondly, the prognosis of these patients is also important. But the follow-up time of this study is too short, which makes this work impossible to implement right now and needs to be investigated in the future.

## Conclusion

Our study suggests that multi-parameter MRI (especially the ADC map)-based radiomics and deep learning models can be used as predictors in differentiating PCa and BPH, thus helping clinicians make better clinical treatment decisions and reducing unnecessary prostate biopsies. The adoption of multi-view radiomics and the eventual combination of deep learning and radiomics methods can effectively improve the diagnostic performance of the constructed models.

## Data availability statement

The raw data supporting the conclusions of this article will be made available by the authors, without undue reservation.

## Ethics statement

The studies involving human participants were reviewed and approved by the institutional review board of Zhongnan Hospital of Wuhan University (no.2020109). The ethics committee waived the requirement of written informed consent for participation.

## Author contributions

CL and MD collected the data, performed the image process, and wrote the manuscript. XZ, JR, and XC performed the statistical analysis and modeling. JC supervised the DL modeling part. FX and HX conceived and supervised this work. All authors contributed to the article and approved the submitted version.
