# Supplementary material for: Multi-view radiomics and deep learning modeling for prostate cancer detection based on multi-parametric MRI
Source: Front Oncol. 2023 Jun 28;13:1198899. doi: 10.3389/fonc.2023.1198899 (PMC10338012; doi:10.3389/fonc.2023.1198899)
Supplement: Supplementary file 1 [file DataSheet_1.pdf]

Supplementary Method

Patients enrollment

As the strict criteria for patient inclusion shown in sFig. 1, we searched the imaging data of all hospitalized patients in the PACS system from January 2018 to December 2021, and found a total of 752 patients admitted to the hospital with mp-MRI examination, of which 311 patients were pathology-proven to have PCa or BPH. Then we applied the 4 exclusion criteria (a-d) to these patients and resulted in 100 PCa cases and 136 BPH cases.

ICC calculation

Twenty samples were randomly selected from all samples for the test of feature reliability and reproducibility. The differences between the features generated by reader 1 and those by reader 2 (inter-observer reliability), as well as the differences between the twice-generated features by reader 1 (intra-observer reproducibility) were all evaluated. Inter- and intra-class correlation coefficients (ICCs) were used to evaluate the agreement of feature extraction. A good agreement was reached when the ICC was greater than 0.8 in this study.

Radiomics Features

Radiomics features were extracted based on the VOI of CT images using pyradiomics software (https://pyradiomics.readthedocs.io/en/latest/). Three categories of features were calculated in this study. The first category (C1) was the original radiomics features, which contained the classes of First order statistics, Shape and texture features while the texture features contained Gray level co-occurrence matrix(GLCM), Gray level Run-length matrix(GLRLM), Gray level size zone matrix(GLSZM), Neighboring Gray Tone Difference Matrix(NGTDM) and Gray Level Dependence Matrix(GLDM). These original radiomics features were calculated based on the original images and the calculation details could be found in pyradiomics docs. (https://pyradiomics.readthedocs.io/en/latest/features.html). Besides, we also extracted the high order features: first processed the original images using different filters or mathematical transformations, then extracted their first order statistics, shape and texture features using pyradiomics method, which was similar to the calculation of original features. Laplacian of Gaussian (LoG, Category 2, C2) and Wavelet (Category 3, C3) filters with different inner parameters were adopted in the generation of high order . Finally, a total of 1561 radiomics features were extracted from the VOI in the images for each patient.

The architecture of the Swin Transformer

As shown in Fig. 2 the end-to-end architecture consists of a patch partition layer, four transformer stages (two, two, six and two transformer blocks in each stage), a global average pooling layer and a

linear layer. In patch partition layer, the three-channel image was split into a non-overlapping patch. Each patch was set as a concatenation of the raw pixel values. In four transformer stages, several transformer blocks with multi-head self-attention layer are applied on these patches to produce hierarchical features, which are the same as ResNet, VGGNet, and other convolutional neural networks. Then the global average pooling layer was used to reduce dimension of extracted hierarchical features by averaging spatial features. Finally, linear layer with softmax function generated risk probability of PCa.

Supplementary Figure

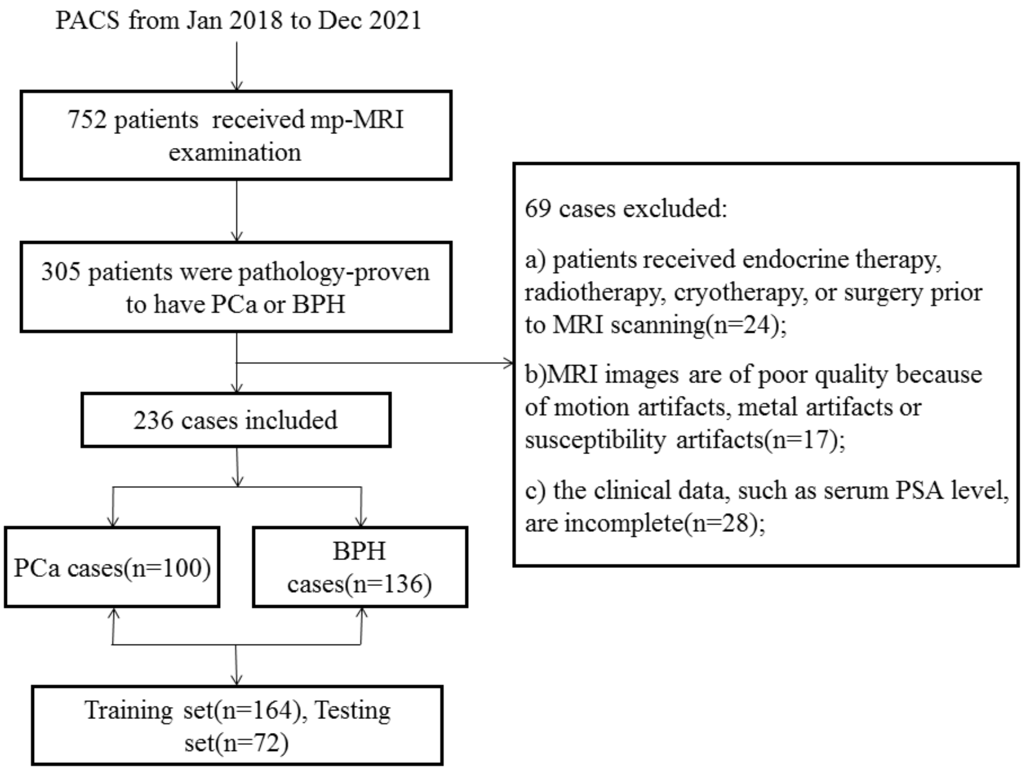

sFigure 1. Flowchart of patient selection of this study. PACS: Picture Archiving and Communications System; PSA: prostate specific antigen.

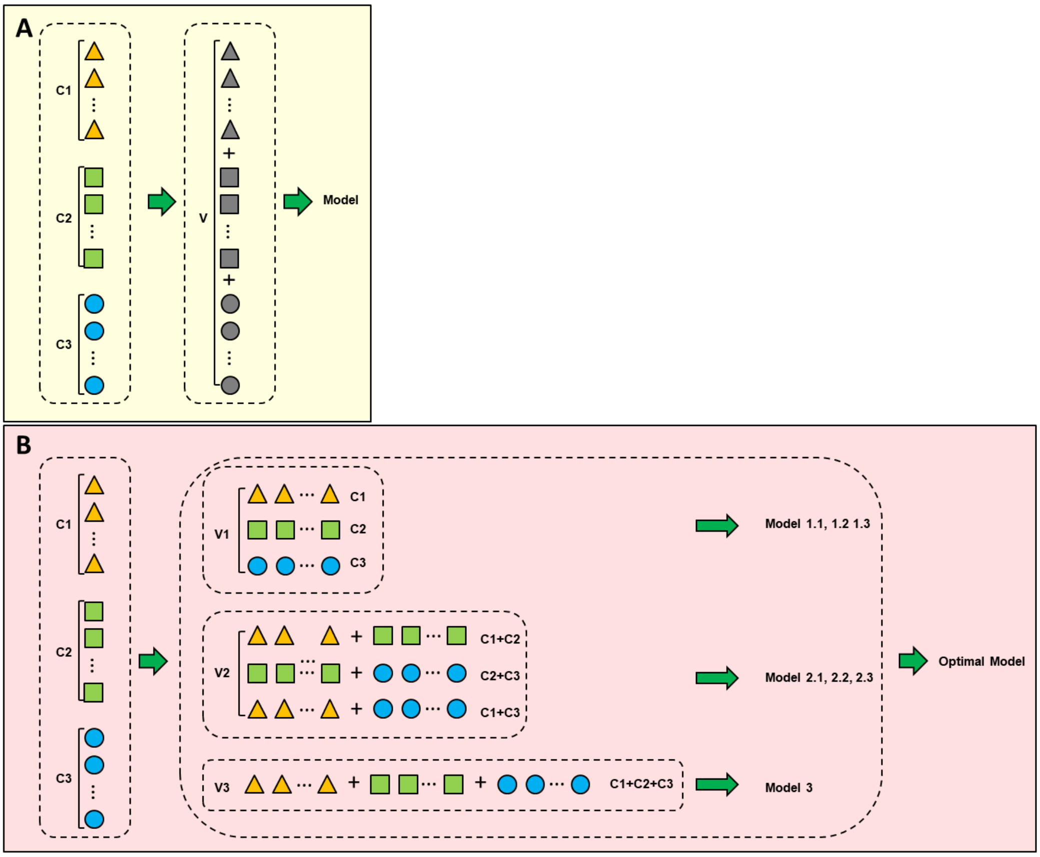

sFigure 2. Comparison of two strategies for handling radiomics features. (A) The single-view strategy. All categories of features are combined without distinction (C1+C2+C3) for subsequent radiomics modeling; (B) The multi-view strategy. Different categories (e.g. C1, C2 and C3) are arbitrarily combined and subsequently used for radiomics modeling. In this strategy, seven possible combinations (C1, C2, C3, C1+C2, C2+C3, C1+C3 and C1+C2+C3) of the features were used to construct the Rdiomics models, respectively. Regardless of which strategy was adopted, radiomics modeling based on radiomics features followed the procedure shown in Fig.1.

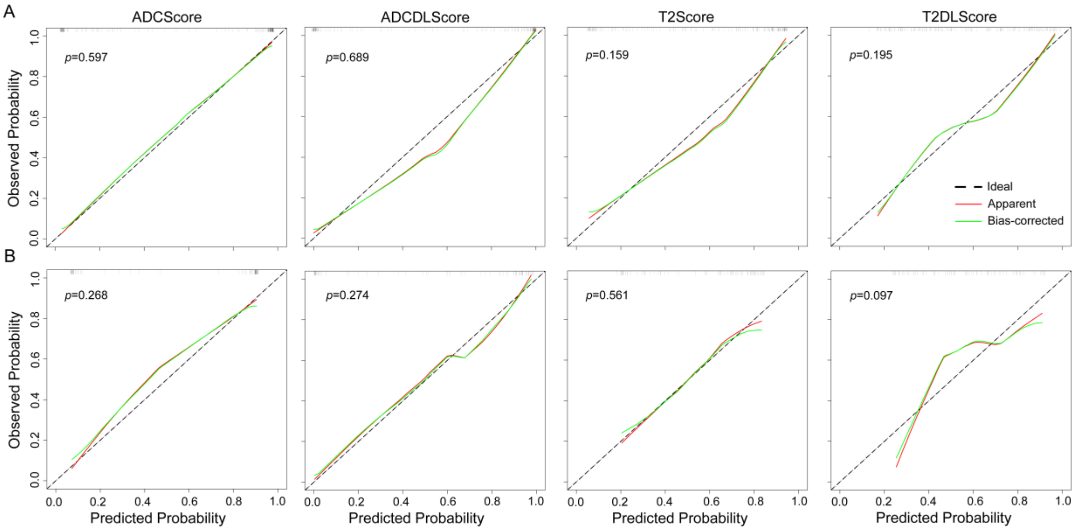

sFigure 3. The calibration curves for the different imaging models. (A) training set (B) test set. From left to right, the calibration curves are for the ADCScore, ADCDLScore, T2Score and T2DLScore model, respectively. The p-value showed the Hosmer–Lemeshow test results for the corresponding models.

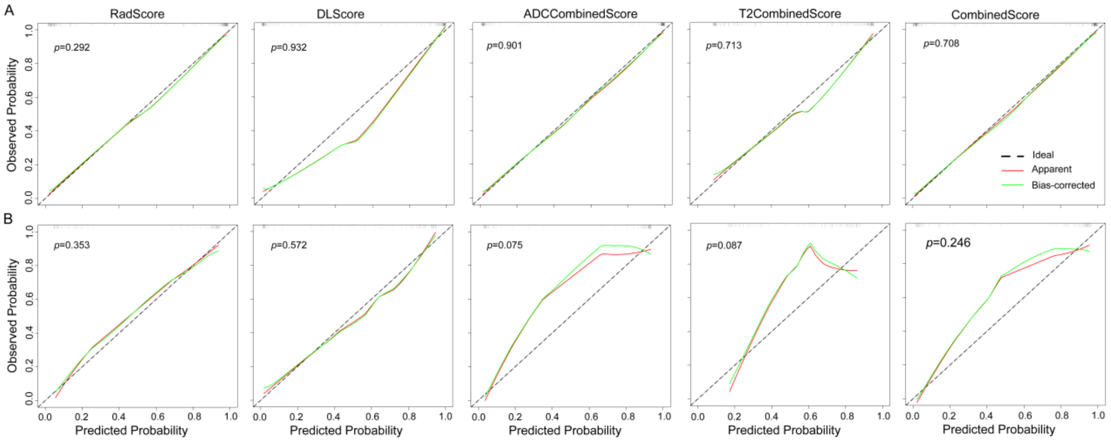

sFigure 4. The calibration curves for different combined models. (A) training set (B) test set. From left to right, the calibration curves are for the RadScore, DLScore, ADCCombinedScore, T2CombinedScore and CombinedScore models, respectively. The p-value showed the Hosmer–Lemeshow test results for the corresponding models.

Supplementary Tables

sTable.1 ADC radiomics features selected for ADCScore model (C1+C3) construction using LASSO method.

index	Feature Name	Weighted.
0	Const	0.3787
1	wavelet.HHL_firstorder_Skewness	-0.0696
2	wavelet.LLH_glszm_GrayLevelNonUniformityNormalized	-0.1380
3	wavelet.LLL_glszm_LargeAreaLowGrayLevelEmphasis	0.0891
4	wavelet.LHH_glcm_ClusterShade	0.0395
5	wavelet.HLH_glcm_ClusterShade	0.1177
6	wavelet.HHH_firstorder_Mean	-0.3535
7	wavelet.LHH_glszm_SizeZoneNonUniformityNormalized	0.0345
8	wavelet.LHH_firstorder_Skewness	-0.1514
9	wavelet.LHH_gldm_SmallDependenceEmphasis	-0.2317
10	wavelet.HLH_glrlm_GrayLevelVariance	0.1543
.11	wavelet.HHH_glszm_GrayLevelVariance	-0.1888
12	original_ngtdm_Contrast	-0.0024
13	wavelet.LLL_firstorder_Skewness	0.2995
14	wavelet.LHH_glrlm_GrayLevelVariance	0.1064
15	wavelet.LLL_glcm_MCC	-0.2143
16	wavelet.HLL_firstorder_Skewness	0.1775
17	wavelet.HLH_gldm_SmallDependenceLowGrayLevelEmphasis	-0.3553
18	wavelet.HLH_glszm_ZonePercentage	-0.3680
19	wavelet.HLH_firstorder_Mean	-0.8639
20	wavelet.LLL_gldm_GrayLevelVariance	-0.1999
21	original_shape_Sphericity	0.7018
22	wavelet.LHH_glszm_ZonePercentage	-0.4352

sTable.2 T2 radiomics features selected for T2Score model (C1+C3) construction using LASSO method.

index	Feature Name	Weighted.
0	Const	0.3626
1	wavelet.HHL_firstorder_Skewness	0.1809
2	wavelet.LLH_glszm_SmallAreaEmphasis	-0.0079
3	wavelet.HHH_glcm_ClusterShade	0.1403
4	wavelet.HHH_glszm_SmallAreaLowGrayLevelEmphasis	0.0659
5	wavelet.HHH_glszm_SizeZoneNonUniformityNormalized	0.1645
6	wavelet.LHL_gldm_SmallDependenceLowGrayLevelEmphasis	-0.0691
7	wavelet.LHL_firstorder_Skewness	-0.0541
8	wavelet.LLL_glszm_SmallAreaLowGrayLevelEmphasis	-0.3546
9	original_ngtdm_Strength	-0.2257
10	wavelet.HHH_glcm_SumSquares	0.0841
11	wavelet.LLL_glcm_Imc2	-0.2170
12	wavelet.HHL_gldm_DependenceNonUniformityNormalized	0.2738
13	wavelet.LLH_firstorder_Median	0.1531
14	wavelet.LHH_glszm_LargeAreaLowGrayLevelEmphasis	0.3394
15	original_shape_Sphericity	0.4559
16	original_firstorder_10Percentile	0.3475
17	wavelet.HHH_gldm_SmallDependenceLowGrayLevelEmphasis	-0.1438

sTable. 3 RadScore model construction using Logistic Regression method

Index	Feature Name	Coef.	*p*-value
0	Const	-4.6127	<0.001
1	T2Score	3.0219	0.005
2	ADCScore	6.0593	<0.001

sTable. 4 DLScore model construction using Logistic Regression method

Index	Feature Name	Coef.	*p*-value
0	Const	-10.2630	<0.001
1	T2DLScore	5.3468	0.024
2	ADCDLScore	15.3253	<0.001

sTable. 5 ADCCombinedScore model construction using Logistic Regression method

index	Feature Name	Coef.	*p*-value
0	Const	-10.1957	<0.001
1	ADCDLScore	13.3721	<0.001
2	ADCScore	5.4442	<0.001

sTable. 6 T2CombinedScore model construction using Logistic Regression method

index	Feature Name	Coef.	*p*-value
0	Const	-3.7576	<0.001
1	T2DLScore	4.2921	0.003
2	T2Score	4.7739	<0.001

sTable. 7 CombinedScore model construction using Logistic Regression method

index	Feature Name	Coef.	*p*-value
0	Const	-10.6130	<0.001
1	T2Score	0.4393	0.800
2	ADCScore	4.8955	0.001
3	T2DLScore	2.3037	0.497
4	ADCDLScore	12.8460	<0.001

sTable 8 radiomics quality score items and evaluation of our study and other prostate radiomics studies.

RQS item number and name	Score of our study	Most common score in the prostate radiomics studies[37]
Item 1: Image protocol quality (0-1)	1	1
Item 2: Multiple segmentation (0-1)	1	0
Item 3: Phantom study	0	0
Item 4: Multiple time points	0	0
Item 5: Feature reduction	3	3
Item 6: Multivariable analysis	0	0
Item 7: Biological correlates	0	0
Item 8: Cut-off analysis	0	0
Item 9: Discrimination statistics	2	2
Item 10: Calibration statistics	1	0
Item 11: Prospective design	0	0
Item 12: Validation	2	2
Item 13: Comparison to "gold standard"	2	0
Item 14: Potential clinical application	2	2
Item 15: Cost-effectiveness analysis	0	0
Item 16: Open science and data	1	0
RQS(Total)	15	10
RQS(%)	41.67	27.78
